# Myeloid deletion and therapeutic activation of AMPK do not alter atherosclerosis in male or female mice

**DOI:** 10.1194/jlr.RA120001040

**Published:** 2020-09-25

**Authors:** Nicholas D. LeBlond, Peyman Ghorbani, Conor O’Dwyer, Nia Ambursley, Julia R. C. Nunes, Tyler K. T. Smith, Natasha A. Trzaskalski, Erin E. Mulvihill, Benoit Viollet, Marc Foretz, Morgan D. Fullerton

**Affiliations:** 1 Department of Biochemistry, Microbiology, and Immunology, Faculty of Medicine, University of Ottawa, Ottawa, Ontario, Canada; 2 Centre for Infection, Immunity and Inflammation, Ottawa, Ontario, Canada; 3 Centre for Catalysis Research and Innovation, Ottawa, Ontario, Canada; 4 University of Ottawa Heart Institute, Ottawa, Ontario, Canada; 5 Université de Paris, Institut Cochin, CNRS, INSERM, Paris, France

**Keywords:** adenosine 5′-monophosphate-activated protein kinase, macrophage, immunometabolism, cholesterol, inflammation

## Abstract

The dysregulation of myeloid-derived cell metabolism can drive atherosclerosis. AMP-activated protein kinase (AMPK) controls various aspects of macrophage dynamics and lipid homeostasis, which are important during atherogenesis. Using LysM-Cre to drive the deletion of both the α1 and α2 catalytic subunits (MacKO), we aimed to clarify the role of myeloid-specific AMPK signaling in male and female mice made acutely atherosclerotic by injection of AAV vector encoding a gain-of-function mutant PCSK9 (PCSK9-AAV) and WD feeding. After 6 weeks of WD feeding, mice received a daily injection of either the AMPK activator A-769662 or a vehicle control for an additional 6 weeks. Following this (12 weeks total), we assessed myeloid cell populations and differences between genotype or sex were not observed. Similarly, aortic sinus plaque size, lipid staining, and necrotic area did not differ in male and female MacKO mice compared with their littermate floxed controls. Moreover, therapeutic intervention with A-769662 showed no treatment effect. There were also no observable differences in the amount of circulating total cholesterol or triglyceride, and only minor differences in the levels of inflammatory cytokines between groups. Finally, CD68+ area and markers of autophagy showed no effect of either lacking AMPK signaling or AMPK activation. Our data suggest that while defined roles for each catalytic AMPK subunit have been identified, complete deletion of myeloid AMPK signaling does not significantly impact atherosclerosis. Additionally, these findings suggest that intervention with the first-generation AMPK activator A-769662 is not able to stem the progression of atherosclerosis.

Atherosclerosis and its downstream cardiovascular complications continue to represent the leading cause of mortality and morbidity in developed countries. While the importance of cells such as vascular smooth muscle and adaptive immune cells in atherosclerosis has recently been highlighted, myeloid-derived cells of the innate immune system, monocytes and macrophages, are a primary driver of disease initiation and progression (1, 2). The hematopoietic differentiation of monocytes is crucial for atherogenesis, and it is now appreciated that modulating monocyte pools can have direct effects on atherosclerotic plaque initiation and progression (3). Moreover, recent lines of evidence have pointed toward the intrinsic metabolic programming of myeloid-derived cells as being a driver of their atherogenic and inflammatory potential (4, 5).

AMP-activated protein kinase (AMPK) is an evolutionarily conserved heterotrimeric serine/threonine kinase that functions to maintain normal metabolic homeostasis by sensing and restoring energy deficits. AMPK acts to limit anabolic and stimulate catabolic programs in the cell. One of the most recognized consequences of AMPK activation is an acute inhibition of both fatty acid and cholesterol synthesis, via inhibiting phosphorylation on acetyl-CoA carboxylase (ACC)1 and ACC2 and 3-hydroxy-3-methyl-glutaryl-CoA reductase, respectively (6, 7). The inhibition of ACC results in the reduction of malonyl-CoA, which in addition to regulating lipogenic flux, relieves the inhibition on mitochondrial fatty acid uptake, leading to an increase in β-oxidation (8). Complementary to this, AMPK, directly and indirectly, stimulates the process of macroautophagy (herein referred to as autophagy), which is critical for processing metabolic substrates, the clearance of damaged or senescent organelles, and, in the context of atherosclerosis, contributes to the mobilization of stored cholesterol in foam cells and reverse cholesterol transport (9).

We and others have shown that AMPK signaling in differentiated cultured macrophages can regulate various aspects of fatty acid and cholesterol metabolism, while simultaneously governing broader metabolic and immune programs (10–14). In the context of atherogenesis, there have been conflicting reports as to the role of myeloid AMPK signaling in the progression of atherosclerosis, though only male mice have been studied (15–17). Moreover, while systemic delivery of AMPK-activating treatments has been shown to reduce lesion size, the delivery of these treatments was over the entire course of the atherosclerosis progression model (18, 19), and the contribution of myeloid AMPK was not addressed. Here, we report that in an acute model of atherogenesis instigated by AAV-delivery of a gain-of-function PCSK9 myeloid-specific disruption of both the α1 and α2 catalytic subunits of AMPK did not alter plaque size or necrotic core in male or female mice. Further to this, when the direct AMPK-activating compound A-769662 was administered to mice in a therapeutic rather than preventative manner, no protection was observed. These unexpected findings lead us to question the specific roles of each catalytic subunit and whether novel allosteric activators of AMPK would offer therapeutic beneficial effects on atherogenesis.

## MATERIALS AND METHODS

### Animal studies

All experiments conducted were in accordance with the Canadian Council of Animal Care and approved by the Animal Care Committee at the University of Ottawa. All mice were housed in ventilated cages at ∼23°C and maintained on a 12/12 h light-dark cycle with ad libitum access to a standard rodent chow (44.2% carbohydrates, 6.2% fat, and 18.6% crude protein; diet T.2018, Harlan Teklad). The generation of AMPKα1^flox^ and AMPKα2^flox^ mice has been previously described (20, 21). AMPKα1^flox^ and AMPKα2^flox^ mice were crossed to obtain double AMPKα1^flox^/α2^flox^ animals and then crossed with lysozyme Cre (LysM-Cre) hemizygous mice (22). Littermate animals were used for these studies by only breeding AMPKα1^flox^/α2^flox^ to AMPKα1^flox^/α2^flox^ LysM-Cre positive (herein referred to as WT and MacKO). All mice described here are on a C57Bl/6J background. At 10 weeks of age, mice were injected intravenously with 2.5 × 10^^10^ genome copies of a mouse PCSK9-AAV (D377Y). This plasmid was obtained from Addgene [plasmid #58376, which was a kindly deposited by Dr. Jacob Bentzon (23)] and packaged in an AAV8 by the Penn Vector Core at the University of Pennsylvania. Immediately following viral infection, mice were placed on a WD (40% kcal from fat, 43% kcal from carbohydrate, 17% kcal from protein, with 0.2% cholesterol; Research Diets D12079) to induce hypercholesterolemia. After 6 weeks, male and female WT and MacKO mice were assigned to groups that received daily intraperitoneal injections of either 30 mg/kg A-769662 or vehicle control (5% DMSO in PBS) for a further 6 weeks (24). Body weights were monitored weekly for the first 6 weeks, then monitored every second day during the last 6 weeks of intervention to ensure accurate dosage for treatment. Blood samples were collected at baseline and biweekly (following a 4–6 h fast). After the completion of the 12 weeks, mice were anesthetized with a ketamine and xylazine mixture (150 mg/kg ketamine and 10 mg/kg xylazine), exsanguinated by cardiac puncture, and perfused with PBS. Tissues of interest were removed and either snap-frozen in liquid nitrogen or placed in 10% formalin. The heart was carefully isolated and placed in 10% formalin and stored at 4°C until processing.

### Cardiac tissue embedding and sectioning

Hearts were fixed in 10% formalin for 48 h at 4°C while changing the formalin solution daily. Hearts were then incubated in sterile 20% sucrose in PBS for 72 h at 4°C while changing the sucrose solution bi-daily. Before embedding, hearts were removed and dried gently of excess sucrose solution. A razor blade was used to cut transversely at roughly the central location of the heart. The upper chambers (containing the aortic sinus) was embedded in a tissue cassette with optimal cutting temperature medium, snap-frozen with liquid nitrogen and stored at −80°C. The aortic sinus was sectioned using a cryostat (Leica CM 1850 cryostat) at 10 μm serial sections at −20°C. Each slide contained (at least) six serial selections collected at 100 μm intervals, spanning at least 600 μm on a total of 10 slides and stored at −80°C.

### Oil Red O staining

Slides containing aortic sinus cross-sections were left to equilibrate at room temperature for 2 min, after which slides were fixed in 10% formalin in a Coplin jar for 10 min at room temperature. While fixation occurred, Oil Red O working solution (30 ml of 0.5% Oil Red O in isopropanol + 20 ml ddH_2_O) was prepared and left to equilibrate for 10 min before being sequentially filtered (initial filtration with a coffee filter followed by filtration through a 0.45 μm syringe filter). Slides were washed once in 60% isopropanol and then stained in Oil Red O working solution for 10 min. Slides were washed twice with 60% isopropanol and then twice with PBS prior to hematoxylin counterstaining. Slides were washed twice with ddH_2_O, and once dry, aqueous mounting medium was added to the slide, and a 1 1/2 cover slide was mounted. Slides were imaged at 20× magnification and tiled together with the EVOS FL auto 2. Atherosclerotic lesions were selected and quantified using ImageJ analysis software (https://imagej.net/Fiji). Each data point is representative of the average plaque area quantified from four sections spanning 400 μm within the aortic sinus from one animal.

### Lesion quantification and analysis

Within the aortic sinus, lesions were stained with H&E and were selected and quantified using ImageJ software. Comparisons were made in tandem using a slide stained with Oil Red O for increased confidence in plaque selection. Quantifications of plaque size were completed for each 10 μm section spanning at least 400 μm of the aortic sinus. The lesion size reported is the average lesion size from four sections spanning the same area within the aortic sinus for each experimental animal.

### Peritoneal macrophage isolation

Mice received an intraperitoneal injection of 1 ml sterile 3% thioglycolate medium 4 days prior to harvest to induce an inflammatory response for the recruitment of immune cells. At harvest, mice were euthanized by cervical dislocation and the abdominal skin was removed to expose the peritoneal cavity. Ten milliliters of ice-cold PBS were slowly injected within the peritoneal cavity through a 20 gauge needle without removing the needle from the peritoneum; PBS and any free-floating peritoneal cells were slowly withdrawn back into the syringe. The 20 gauge needle was removed, and cells were slowly ejected into a sterile 50 ml conical tube and placed on ice until all isolations were completed. The cells were then centrifuged at 95 *g* for 5 min at 4°C; the supernatant was removed; and the cell pellet was gently resuspended in 10 ml DMEM (supplemented with 10% FBS and 100 U/ml penicillin and streptomycin). Cells were seeded onto one to three 100 mm cell culture dishes and left to adhere for 16 h, then washed twice with PBS, gently scraped in complete DMEM, and seeded onto 34.8 mm plates.

### Immunoblotting

Peritoneal cells were washed twice with ice-cold PBS, and then cells were scrapped in 60–120 μl cell lysis buffer [50 mM Tris-HCl (pH 7.5), 150 mM NaCl, 1 mM EDTA, 0.5% Triton X-100, 0.5% NP-40, 100 μM Na_3_VO_4_, supplemented with protease inhibitor cocktail]. All protein was quantified (via bicinchoninic acid assay; Pierce™), equalized, and 4× loading dye was added prior to denaturing the protein by boiling at 95°C for 5 min. Protein samples were loaded and electrophoresed onto duplicate 8% SDS-PAGE gels (15–20 μg/well) for the determination of phosphorylated proteins along with their respective total amounts. Each gel was transferred onto PVDF membranes using the Trans-Blot Turbo system (25 V, 2.5 mps, for 18 min; Bio-Rad). Membranes were blocked in 5% BSA in TBST (w/v) with gentle rocking (Rocker 25; Mendel) for 1 h at room temperature, then incubated in the primary antibody of interest overnight at 4°C. The following day, membranes were washed four times in TBST at room temperature (5 min per wash), and membranes were incubated with anti-rabbit HRP-conjugated secondary antibody for 1 h at room temperature with gentle rocking and washed four more times in TBST. Clarity ECL substrate mix was added to membranes, which were imaged using the ImageQuant LAS4000 (GE) system. Quantification of total and relative luminescence for all proteins was done using ImageJ analysis software.

### Immunofluorescent labeling

Slides containing aortic sinus cross-sections were left to equilibrate to room temperature for 2 min and fixed in 2% PFA for 20 min. Following fixation, a hydrophobic marker (ImmunoPen; Millipore) was used to create barriers around all sections present on the slides. Slides were washed once in PBS and then blocked/permeabilized with PBS containing 5% fatty acid-free BSA, 0.2% Triton X-100, and Tween-20. Samples were washed with PBS then co-stained with anti-mouse CD68-Alexa-647 (BioLegend) and p62-FITC (NovusBio) for 1 h in antibody solution (2% BSA, 0.1% Triton X-100, and Tween-20 in PBS) at room temperature in a dehumidified chamber (shielded from light). Samples were washed twice with PBS and then incubated with anti-FITC Alexa-488-conjugated secondary antibody (Invitrogen) for 1 h at room temperature in a dehumidified chamber. Samples were washed with PBS and then incubated in 300 nM DAPI (Thermo Fisher Scientific) for 5 min at room temperature shielded from light. Samples were washed twice with PBS; ProLong Gold antifade reagent (Invitrogen) was added to slides that were then mounted with 1 1/2 coverslip (Corning). Fluorescent microscopy images were taken using the EVOS FL Auto 2 Imaging System using a 20× magnification. All fluorescent images were processed and quantified using ImageJ analysis software.

### Plaque CD68 composition

First, we defined and selected lesions from immunofluorescent images using ImageJ software. Selected lesion areas were quantified for specific regional analysis. The lesions underwent thresholding to measure positive areas of fluorescence (where CD68 is expressed) within the plaque being defined as CD68+ area. The proportion of lesion area that was positive for CD68 expression was calculated as follows: (CD68+ lesion area/total lesion area) × 100 = %CD68+ plaque area. Each reported value was the average of three sections within the aortic sinus of one animal, the same regions were quantified and compared for all animals.

### Lesion p62 staining

First, we defined and selected lesions from immunofluorescent images using ImageJ software. Within selected regions, we quantified single-channel fluorescent intensity values corresponding to p62 expression within the whole lesion and within CD68+ areas. We analyzed the mean, median, and modal fluorescent intensities for the combined plaque within a section, but only the mean fluorescent intensities were reported. Each reported value was the average of three sections within the aortic sinus of one animal; the same regions were quantified and compared for all animals.

### Serum lipid determination

Mice were fasted for 4 h prior to tail bleed (or cardiac puncture at experimental endpoint). Blood was left at room temperature for 30 min to allow it to clot and then centrifuged at 857 *g* for 10 min to allow phase separation. The top opaque yellow phase (serum) was collected, aliquoted, and stored at −20°C. Serum was thawed and analyzed accordingly by the commercially available Infinity-cholesterol™ kit (Thermo Fisher Scientific) and a triglyceride colorimetric assay kit (Cayman Chemical) as per the manufacturer’s instructions.

### Serum inflammatory cytokine analysis

Serum cytokines were quantified using the Meso Scale Discovery Pro-Inflammatory Panel 1 (mouse) kit (MSD, Gaithersburg, MD) assay following the manufacturer’s protocol. Serum C–C motif chemokine ligand 2 (CCL2) was determined by ELISA (R&D Systems), as per the manufacturer’s instructions.

#### Flow staining of immune populations.

Splenocytes were isolated by passing through nylon mesh strainers. Bone marrow cells were centrifuged from femurs and tibiae with cut epiphyses as previously described (25). Blood was obtained via cardiac puncture and collected in EDTA-coated tubes. Erythrocytes were lysed in red blood cell lysis buffer (155 mM NH_4_Cl, 10 mM NaHCO_3_, 10 mM EDTA in water) with blood samples treated in 10 ml twice and splenocytes and bone marrow cells treated in 1 ml once. Cells were stained with Zombie Aqua (BioLegend) for 30 min on ice with anti-CD16/CD32 (unless included in surface staining) in PBS. Surface marker staining was performed in PBS + 0.5% BSA/2 mM EDTA/0.05% NaN3 (PBA-E) for 20 min on ice. Surface antibodies (from BioLegend unless otherwise specified) included: biotin-conjugated lineage cocktail (anti-B220, anti-Ter119, anti-CD11b, anti-Gr-1, anti-CD3ε; #133307), anti-Sca-1-PacificBlue (#108119), anti-c-Kit-PE/Cy7 (#105813), anti-CD34-eFluor660 (Thermo Fisher, #50-0341-82), anti-CD16/32-Brilliant Violet 711 (#101337), anti-CD115-PE (#135505), anti-Ly-6G-FITC (#127605), and anti-Ly-6C-PE/Cy7 (#128017). Secondary stain with streptavidin-Alexa Fluor 488 (BioLegend, 405235) for 20 min on ice was done where necessary. Cells were fixed in 1–2% PFA in PBS for 15 min on ice and stored in PBA-E until acquired on an LSRFortessa cytometer (BD Biosciences). Analysis was performed using FlowJo VX (Treestar Inc.).

### Statistics

For atherosclerosis immunofluorescent and biochemical analyses, all comparisons made between genotype and treatment, both within and between biological sex, were made using two-way ANOVA with a Tukey test for multiple comparisons (GraphPad Prism 7).

## RESULTS

### Deletion of myeloid AMPK signaling does not alter monocyte populations

While AMPKα1 is the predominant isoform expressed in hematopoietic cells (12, 26), myeloid deficiency of either AMPKα1 or AMPKα2 has been shown to alter atherosclerosis (15–17). Moreover, these studies crossed their respective AMPK-deficient mouse models onto the atherogenic *LDLr*- or *ApoE*-deficient background. To completely disrupt AMPK signaling, we generated mice lacking all AMPK signaling in myeloid cells (AMPKα1 and AMPKα2 deletion driven by LysM expression of Cre recombinase), which was confirmed by assessing AMPK-specific signaling to its downstream target, ACC, in elicited peritoneal macrophages from floxed littermate controls (WT) and MacKO mice. As expected, basal AMPK signaling to ACC was almost completely absent in MacKO cells, with no effect of the AMPK activator, A-769662 (supplemental Fig. S1). As signaling to ACC has long been shown to be AMPK-specific (27), the residual signal is likely due to the presence of nonmyeloid cells. Importantly, basal AMPK signaling in the liver was unaffected (supplemental Fig. S1).

**Fig. 1.**
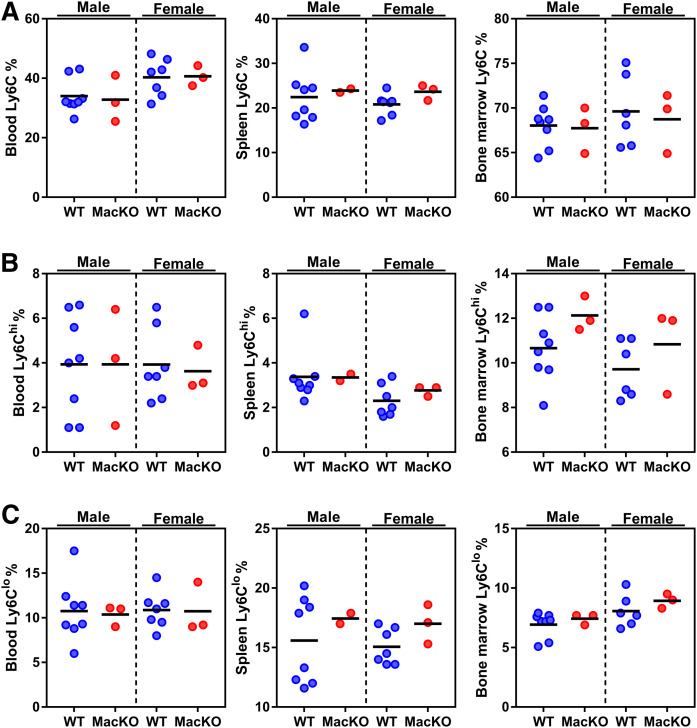
Myeloid AMPK does not alter myelopoiesis. WT and MacKO male and female mice were injected with the PCSK9-AAV and fed a WD for 12 weeks. The expression of total Ly6C (A), Ly6C^hi^ (B), and Ly6C^lo^ (C) populations from the blood, spleen, and bone marrow quantified. Each data point represents the mean value from one animal (n = 2–8/group). One splenic sample from the male MacKO group was removed due to poor cell staining.

To circumvent genetic mouse models of atherosclerosis, we used a single intravenous injection of a well-characterized gain-of-function (D377Y) mouse PCSK9-AAV followed by WD feeding to induce rapid hypercholesterolemia to drive atherogenesis for 12 weeks (supplemental Fig. S2) (23). Monocyte-derived macrophages are a key cell type in atherosclerosis, and myeloid cell maturation and differentiation, which begins in the bone marrow via myelopoiesis, can be a driver of atherosclerosis. Moreover, myelopoiesis is augmented by WD-induced hypercholesterolemia in both mice and humans (28). In a preliminary cohort of male and female WT and MacKO mice, we first aimed to determine whether there were any basal genotype differences in myeloid cell populations. Using Ly6C, CD115, and Ly6G as myeloid cell markers, we observed no trend differentiating circulating, splenic, or bone marrow populations, either between genotypes or between males and females (**Fig. 1A**, supplemental Fig. S3). Moreover, the presence or absence of myeloid AMPK did not skew the levels of Ly6C^hi^ or Ly6C^lo^ populations, which are recognized as monocytes prone (Ly6C^hi^) or resistant (Ly6C^lo^) to effector or patrolling functions, respectively (2) (Fig. 1B, C). To investigate myeloid differentiation, we determined that bone marrow Lin^−^Sca-1^+^c-Kit^+^ cells, as well as bone marrow and splenic populations of multipotent progenitors, common myeloid progenitors, granulocyte-macrophage progenitors, and megakaryocyte-erythroid progenitors were also unaltered (data not shown). However, these studies remain preliminary and underpowered; therefore, future studies are warranted before conclusions can be made as to the role that myeloid AMPK plays in modulating these immune populations.

### Myeloid AMPK signaling has minimal impact on the atherosclerotic plaque

**Fig. 2.**
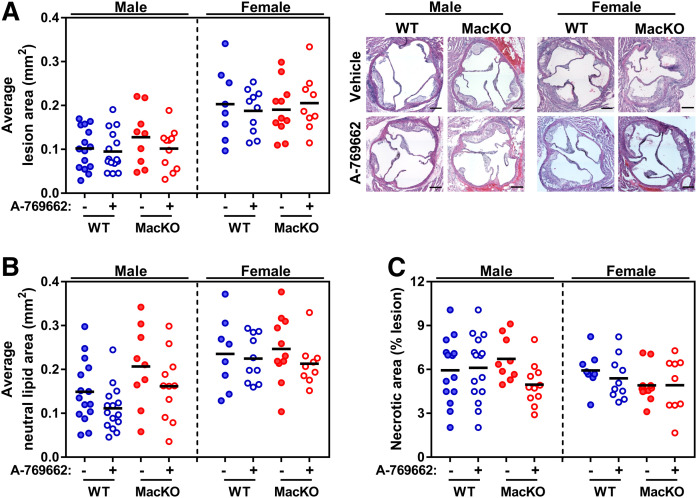
Myeloid AMPK signaling does not alter the progression of atherosclerosis in male and female mice. WT and MacKO male and female mice were injected with the PCSK9-AAV and fed a WD for 12 weeks, with half of each group receiving either 30 mg/kg A-769662 or vehicle control for the final 6 weeks. A: Average lesion area quantified within the aortic sinus (representative H&E-stained images are included). B: Quantification of the average Oil Red O-containing areas within the aortic sinus. C: Lesion necrotic area expressed as percent lesion area. A–C: Selection and quantifications were performed with ImageJ software of H&E-stained (A, C) and Oil Red O-stained (B) slides as listed in the Materials and Methods. Scale bar represents 100 μm. Each data point represents the mean value from one animal (n = 7–16/group).

Male and female mice of both genotypes gained weight as expected when fed a WD. After 6 weeks, each group was divided and received daily injections with either vehicle control or the first-generation AMPK activator A-769662 (30 mg/kg) for the last half of the 12 week intervention. Both male and female mice, independent of myeloid AMPK signaling, experienced a reduction in body weight, as has been previously documented (24) (supplemental Fig. S4). At the completion of the 12 week study, the atherosclerotic lesion area was quantified from the aortic sinus. In male and female mice, there were no differences in lesion size between genotypes. In addition, treatment with A-769662 as an intervening therapy had no significant effect on plaque size (**Fig. 2A**). When plaque sections were stained with Oil Red O to quantify neutral lipid-rich areas, there were no significant differences between groups (Fig. 2B). Consistently, the amount of necrotic area was also not changed by the presence of myeloid AMPK, treatment with A-769662 or, between sex (Fig. 2C, supplemental Fig. S5). These data suggest that in the early stages of PCSK9-induced atherosclerosis, myeloid AMPK signaling does not regulate total lesion area, lipid content, or necrotic area.

### Myeloid AMPK and systemic activation do not affect total circulating lipid levels

**Fig. 3.**
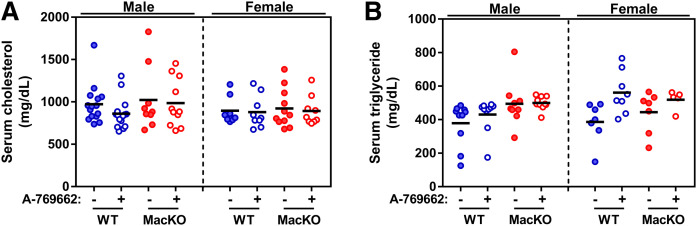
Myeloid AMPK signaling does not change total levels of circulating lipids. Following WD feeding, serum was collected to assess total circulating cholesterol (A) and total circulating triglycerides (B). Each data point represents the value from one animal (n = 5–16/group).

The liver is a significant regulator of whole-body lipid metabolism and can dictate levels of lipoprotein-associated cholesterol and triglyceride in the circulation. While disruption of AMPK was restricted to myeloid cells, there was the potential that LysM-mediated deletion would occur in liver-resident macrophages (Kupffer cells), which could affect circulating lipid levels (29). Moreover, our experimental design made use of the systemic delivery of A-769662, which has well-known effects on lipid metabolism (24, 27). Independent of genotype, treatment, or sex, there were no differences in the levels of circulating total cholesterol or triglyceride (**Fig. 3A**, B).

### Myeloid AMPK does not regulate systemic inflammation during atherosclerosis

**Fig. 4.**
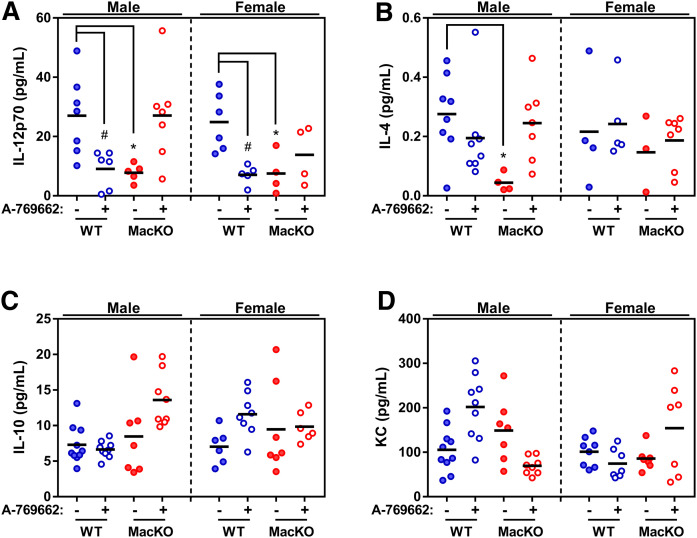
Myeloid AMPK signaling slightly alters systemic inflammation during the progression of atherosclerosis. Following WD feeding, serum was collected to assess circulating inflammatory cytokines IL-12p70 (A), IL-4 (B), IL-10 (C), and KC (D). Each data point represents the value from one animal (n = 4–10/group), where * represents *P* < 0.05 between genotypes and # is *P* < 0.05 between treatments as calculated by a two-way ANOVA.

Metabolism and inflammation are recognized as important drivers of atherogenesis. Given the known role for AMPK in modulating both macrophage metabolism and immune programs, we next assessed the systemic (circulating) levels of inflammatory cytokines (IL-10, IL-12p70, IL-6, KC, TNFα, IL-1β, IFNγ, IL-2, IL-4, and IL-5) (**Fig. 4**, supplemental Fig. S6). In male mice, at the time of tissue harvest and blood collection, circulating levels of IL-12p70 and IL-4 were significantly lower in MacKO compared with control mice. When comparing vehicle and A-769662 treatment, IL-12p70 levels decreased in WT-treated but increased in MacKO-treated mice (Fig. 3A–D). In addition, levels of KC (mouse IL-8), were significantly increased in A-769662-treated WT mice, but lower in MacKO mice treated with the AMPK activator. While levels of other cytokines went unchanged, IL-12p70, IL-4, and IL-10 were all augmented by A-769662 treatment in mice that were deficient for myeloid AMPK, suggesting non-AMPK or nonmyeloid mechanisms. In female animals, only IL-12p70 differed significantly, such that levels in MacKO mice were lower compared to WT.

Previous work has demonstrated that preventative AMPK activation (i.e., AMPK activator treatment at the start of dietary initiation of atherosclerosis) was associated with decreased levels of the circulating chemokine CCL2 (18, 30). Consistent with a lack of difference in total plaque area, circulating CCL2 was not different between any of the groups (supplemental Fig. S7).

### Myeloid AMPK signaling does not alter the amount of CD68+ cells

**Fig. 5.**
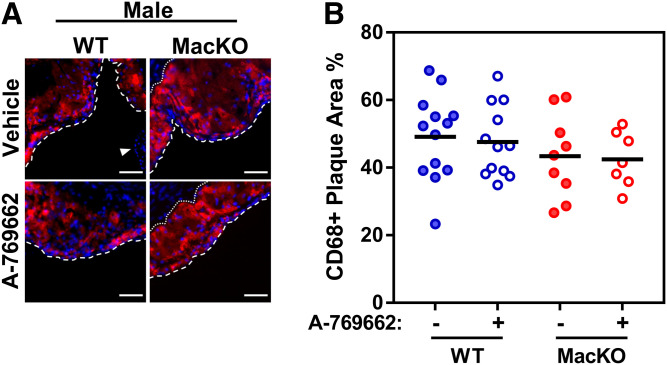
Myeloid AMPK signaling does not regulate the proportion of CD68-positive cells within the lesion. A: Representative images for immunofluorescent labeled lesions of male mice (CD68, red; DAPI, blue; where the luminal border of the lesion is denoted by white dashed and dotted line). B: Quantification of CD68+ area as a percent of the total lesion area in lesions of male mice. Scale bar represents 50 μm. Each data point represents the mean value from one animal (n = 7–13/group).

Deletion of myeloid AMPK on an *ApoE*-deficient (16) or *LDLr*-deficient (17) background resulted in less and more markers of macrophage accumulation, respectively. To interrogate this in our model, we used CD68 as a general marker of macrophage-like cells within atherosclerotic lesions because it is now well-established that vascular smooth muscle cells can adopt CD68 expression as atherogenesis progresses (31). Similar to the results above, we observed no difference in aortic lesion area between male WT and MacKO mice. We detected no difference in the amount of lesion CD68+ expression between groups, regardless of genotype or treatment when normalized to total area (**Fig. 5A**, B).

### Myeloid AMPK signaling does not alter markers of autophagy

**Fig. 6.**
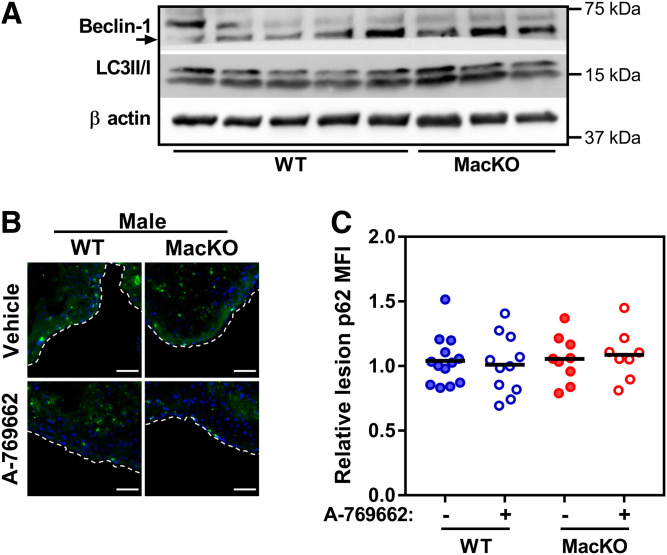
Myeloid AMPK signaling does not influence markers of lesion autophagy. A: Whole aortic protein lysate from WT and MacKO (male) mice was probed for the expression of Beclin-1 and LC3II/1. B: Representative images for immunofluorescent labeled lesions of male mice (p62, green; DAPI, blue; where the luminal border of the lesion is denoted by white dashed line). C: Quantification of the relative mean fluorescent intensity for p62 within the lesions of male mice. Scale bar represents 50 μm. Each data point represents the mean value from one animal (n = 8–13/group).

We next aimed to determine whether the disruption or activation of AMPK signaling in myeloid cells influenced markers of autophagy within the plaque environment. We began by probing for the expression of autophagy markers Beclin-1 and microtubule-associated proteins 1A/1B light chain 3B (LC3II/I) in whole aortic lysates of male WT and MacKO mice; however, no basal genotype differences were observed (**Fig. 6A**). We next stained the atherosclerotic lesion for p62 (also known as SQSTM1), an essential chaperone protein that is processed in an autophagic-dependent matter and serves as a marker of defective autophagy (32). The average level of plaque-associated p62 was unaffected by the presence or absence of myeloid AMPK, and treatment with A-769662 had no effect on the levels of p62 staining within lesions (Fig. 6B, C) in male mice. Additionally, we did not observe any alterations in p62 expression within CD68+ areas (supplemental Fig. S8).

## DISCUSSION

The progression of atherosclerosis is causative in the development of cardiovascular disease. While mechanistic understanding has translated to effective frontline therapies against cardiovascular disease, incidence and societal burden remain high. Numerous pathways and cell types are engaged at the onset of atherosclerosis; however, metabolic and cell differentiation pathways of myeloid cells are critical (2). Because AMPK is known to regulate multiple immunometabolic programs (8, 33), we sought to address the importance of AMPK signaling in myeloid cells during the progression of atherosclerosis. Moreover, building on evidence that chronic preventative treatment of WD-fed *ApoE*-deficient mice with AMPK activators (including A-769662) protected against lesion development (18, 19), we asked whether therapeutic intervention with systemic AMPK activation protects against atherogenesis in a myeloid AMPK-dependent manner.

To avoid lengthy breeding schemes and the constitutive nature of *Ldlr*^−/−^ and *ApoE*^−/−^ mouse models, we administered a validated gain-of-function (D377Y) PCSK9-AAV, which was driven by a liver-specific promoter (23). While we aimed to provide clarity as to the role of myeloid AMPK in atherosclerosis, in our model, there were no statistical differences in plaque, lipid, or necrotic area between mice that had or did not have myeloid AMPK signaling or mice that were treated with or without the direct AMPK activator A-769662 in male and female mice. However, we observed that in male mice only, myeloid AMPK signaling showed a trend toward restraining the lipid content of atherosclerotic plaque in an AMPK-dependent manner (Fig. 2B). Similarly, we found that intervention with an AMPK activator tended to stem plaque lipid accumulation and that myeloid AMPK signaling seemed to be required. There has been a shift toward the use of a PCSK9-overexpressing AAV to drive atherosclerosis in genetic mouse models; however, this new method makes it unfortunately difficult to compare previous studies.

There is no clear answer to the question as to whether myeloid AMPK plays a beneficial or detrimental role in atherogenesis. Our results in a different model of atherosclerosis neither support nor refute previous studies, which were themselves contradictory. When myeloid AMPKα1 deletion was driven by LysM expression on a *Ldlr*-deficient background, these mice had more atherosclerotic lesion and plaque area, higher circulating lipids, more Ly6C^hi^ monocytes, and more pro-inflammatory markers in the aorta (17). Opposing this, myeloid AMPKα1 or AMPKα2 deletion via LysM-mediated Cre expression on an *ApoE*-deficient background resulted in smaller atherosclerotic lesions. This was attributed to an AMPKα1-dependent effect on regulating monocyte-to-macrophage differentiation and autophagy (16) or an AMPKα2-dependent effect on DNA methylation and altered expression of matrix metalloproteinases (15), respectively. The phenotype of the myeloid AMPKα2-deficient mice is interesting given that the predominant isoform in myeloid cells was shown to be AMPKα1 (12, 34). Given this, it might be expected that a compensatory upregulation of the AMPKα2 subunit might occur during AMPKα1 deficiency; however, the opposite is not as intuitive.

Rather than deal with isoform-specific contributions, we chose to delete both catalytic subunits to best characterize the significance of total myeloid AMPK signaling. One potential limitation to using LysM-Cre as a driver of myeloid-specific disruption is the role of AMPK signaling in nonmyeloid hematopoietic cells, which may compensate or substitute for disrupted signaling and potentially mask myeloid contributions to atherogenesis.

The largest discrepancy between studies that show a protective role and those that show a detrimental role for myeloid AMPK is genetic background (LDLr vs. ApoE). Atherosclerosis is lessened in response to myeloid AMPKα1 and AMPKα2 deletion only when on an *ApoE*-deficient background (15, 16). Moreover, AMPKα1/ApoE mice had no difference in serum cholesterol and AMPKα2/ApoE mice had higher total cholesterol levels compared with floxed/ApoE control mice, suggesting that these effects were independent of circulating cholesterol. Contrary to this, circulating cholesterol levels in AMPKα1/LDLr mice were positively correlated with lesion size and were increased when myeloid AMPKα1 was absent (17). In our model, we did not observe any differences in total levels of circulating lipids, which was consistent with the similarity in total lesion area in all groups.

There remains the potential that regulatory differences in AMPK signaling exist in myeloid cells that have or do not have LDLr or ApoE. Aldehyde dehydrogenase 2 (ALDH2) disruption was atheroprotective on an *ApoE*-deficient background but atherogenic on a *Ldlr*-deficient background (35). The presence of the LDLr blocks AMPK from phosphorylating ALDH2, which leads to histone deacetylase 3 (HDAC3)-mediated downregulation of lysosomal programs and increased foam cell formation (35). Two considerations are important when applying this to our current findings. First, myeloid deletion of AMPK signaling would remove the negative signaling to HDAC3 and serve to enhance programs that lower atherogenesis. Second, in our PCSK9-induced model, whether circulating PCSK9 has LDLr-suppressive effects in nonhepatic tissues (specifically circulating monocytes and plaque macrophages) remains unknown, making any interpretation of myeloid AMPK signaling working via LDLr premature.

AMPK signaling has been shown to induce autophagy directly via activating phosphorylation of Unc-51-like autophagy activating kinase 1, Beclin-1, and VPS34, and indirectly via inhibition of the mechanistic target of rapamycin complex 1, which itself is regulated indirectly by AMPK-mediated phosphorylation of tuberous sclerosis complex 2 and/or regulatory-associated protein of mTOR (36). It is now well-established that autophagy is athero-protective due to its clearance of cellular debris, sequestration of defective organelles, and liberation of free cholesterol for the purpose of cholesterol efflux (37). To assess one marker of lesion autophagy, we stained lesions for p62, the accumulation of which is associated with defective autophagy. Despite initial predictions, there were no genotype or treatment effects on the expression or localization of p62 within the developed lesions. With a trending decrease in lesion lipid content, it remains entirely plausible that the effects of AMPK signaling are linked to its regulation of autophagy; however, this was not captured by p62 staining. In isolated macrophages, we and others have demonstrated that in addition to an acute regulation of autophagy induction, AMPK exerts a level of transcriptional regulation via direct and indirect modulation of transcription factor EB and lysosomal programs (38–40).

Previous reports show that genetic deletion of myeloid AMPK primes monocytes toward a more pro-inflammatory phenotype, elevating proportions of Ly6C^hi^ to Ly6C^lo^ monocytes in the circulation or in the peritoneum in response to thioglycollate (17). In keeping with this, mice treated systemically and chronically with A-769662 had a similar response and fewer Ly6C^hi^ cells (18). In the present study, with all myeloid AMPK signaling disrupted, we observed absolutely no effect on circulating, splenic, or bone marrow populations of myeloid or precursor cells. This was consistent with the lack of difference regarding plaque size but differed with results from *ApoE* and *Ldlr*-deficient models (15–17). As a correlate of immune infiltration into the plaque, we used CD68 as a marker of macrophages and macrophage-like cells, although CD68 is also known to be expressed on dendritic cells, neutrophils, and smooth muscle cells (31). Unexpectedly, we observed no differences in the apparent proportion of lesion-associated CD68+ cells. While chronic A-769662 treatment in *ApoE*-deficient mice resulted in fewer Ly6C^hi^ monocytes in conjunction with decreased C-C chemokine receptor type 2 (*Ccr2*) expression, this was recently expanded by the observation that in response to acute fasting or acute (4 h) AMPK activation with A-769662, Ly6C^hi^ monocytes are dramatically reduced due to lower levels of CCL2, the ligand for CCR2 (30). In our model of complete myeloid AMPK deletion, we see that circulating CCL2 is not altered. Importantly, while the effects of acute fasting and AMPK activation were attributed to a hepatic AMPK signaling, we did not observe any differences in CCL2 levels following systemic AMPK activation.

There have been an impressive number of studies that have aimed to assess the contribution of AMPK toward atherosclerosis. Whole-body (16, 41, 42), vascular smooth muscle (43, 44), endothelial (43, 45), and myeloid-specific models on either *ApoE*- or *Ldlr*-deficient backgrounds (15–17) have been used in conjunction with either AMPKα1 or AMPKα2 KO models. Despite this, to our knowledge, female mice have rarely (if ever) been assessed. Our results suggest that while there are no significant differences between male and female MacKO mice and WT littermates, there is a potential sex-specific trend whereby AMPK signaling may act to lower lesion lipid content in male but not female mice. Sex-specific responses will continue to be an essential consideration in any future work. Moreover, an essential difference between our study and past published work was that we undertook the deletion of both catalytic subunits of AMPK in myeloid cells. One potential interpretation is that the effects (beneficial or detrimental) observed in models of single AMPK deletion may be lost due to unrecognized differential signaling between these two isoforms, either in myeloid or other immune cells. This point highlights the inherent limitations in KO models of important metabolic regulators and the need for more pointed models to tease out the importance of specific signaling nodes.

We feel it is important to address some of the limitations and caveats of our study. *1*) We began by addressing how myeloid AMPK deficiency may alter immune populations; however, we did not assess mice following the intervention with A-769662. While this may have illuminated differences in monocyte differentiation, there were no statistical differences in atherosclerosis. *2*) Also, we chose to inject our WT and MacKO mice with a gain-of-function PCSK9-AAV to induce atherosclerosis (2.5 × 10^^10^ viral particles per mouse), which, at this concentration when coupled with the WD-feeding, resulted in levels of circulating cholesterol and plaque sizes that were lower than what is typically observed in *LDLr*-deficient mice (23). Though 12 weeks of atherosclerosis progression should be sufficient for genotype differences to emerge, it may be possible that differences could have been observed earlier or later. Moreover, although treatment with A-769662 daily during atherogenesis was shown to be protective, intervention at the same dose was ineffective in stemming disease progression and suggests that AMPK-activation may be necessary from the start of atherogenesis to provide a therapeutic benefit. However, this does not rule out the possibility that second-generation allosteric AMPK activators (PF-739, PF-249, or MK-8722), which are more potent and specific AMPK activators, will not show a therapeutic benefit during an intervention. This will be an important consideration for future work.

Our study is the first to question the role of both AMPKα1 and AMPKα2 in myeloid cells and suggests that the presence and activation of myeloid AMPK signaling does not impact atherosclerotic plaque size in the aortic root, independent of sex. Importantly, there were no changes in myeloid cell numbers, circulating lipid levels, or systemic inflammation. While we have taken yet another approach to assessing the contribution of AMPK to atherogenesis (PCSK9-induced atherosclerosis and deletion of both myeloid AMPKα1 and AMPKα2), future work should concentrate on dissecting the specific molecular and metabolic pathways by which this important regulator may act. Moving forward, rather than the traditional sledghammer knockout approach, targeted phosphorylation knock-in mouse models of known AMPK substrates will be the only way to specifically interrogate the mechanism(s) by which AMPK-regulated pathways like autophagy and lipid metabolism affect atherosclerosis and other cardiometabolic diseases.

### Data availability

The data that support the findings of this study are all listed in the article and available from the corresponding author upon reasonable request.

## Supplementary Material

Supplemental Data
