# Supplementary material for: Myeloid deletion and therapeutic activation of AMPK do not alter atherosclerosis in male or female mice
Source: J Lipid Res. 2020 Sep 25;61(12):1697–706. doi: 10.1194/jlr.RA120001040 (PMC7707174; doi:10.1194/jlr.RA120001040)
Supplement: Supplemental Data [file supp_61_12_1697__index.html]

Myeloid deletion and therapeutic activation of AMPK do not alter atherosclerosis in male or female mice — Myeloid AMPK signaling in atherosclerosis — Myeloid deletion and therapeutic activation of AMPK do not alter atherosclerosis in male or female mice — Supplemental Data 

# Myeloid deletion and therapeutic activation of AMPK do not alter atherosclerosis in male or female mice

## Supplemental Data

- Supplemental Figures - Supplemental figures 1-8
