# Supplementary material for: Myeloid deletion and therapeutic activation of AMPK do not alter atherosclerosis in male or female mice
Source: J Lipid Res. 2020 Sep 25;61(12):1697–706. doi: 10.1194/jlr.RA120001040 (PMC7707174; doi:10.1194/jlr.RA120001040)
Supplement: Supplemental Data [file supp_RA120001040_162404_2_supp_601175_qh2r35.pdf]

SUPPLEMENTARY MATERIAL:

Myeloid deletion and therapeutic activation of AMPK do not alter atherosclerosis in male or female mice

Nicholas D. LeBlond[1,2,3], Peyman Ghorbani[1,2,3], Conor O'Dwyer[1,2,3], Nia Abursley[1], Julia R. C. Nunes[1,2,3], Tyler K.T. Smith[1,2,3], Natasha A. Trzaskalski[1,2,4], Erin E. Mulvihill[1,2,4], Benoit Viollet[5], Marc Foretz[5] and Morgan D. Fullerton[1,2,3]*

[1]Department of Biochemistry, Microbiology and Immunology, Faculty of Medicine, University of Ottawa, Ottawa, ON, K1H 8M5, Canada

[2]University of Ottawa Centre for Infection, Immunity and Inflammation, Ottawa ON K1H 8M5, Canada

[3]Centre for Catalysis Research and Innovation, Ottawa ON K1H 8M5, Canada

[4]University of Ottawa Heart Institute, Ottawa, ON K1Y 4W7, Canada

[5]Université de Paris, Institut Cochin, CNRS, INSERM, F-75014 Paris, France

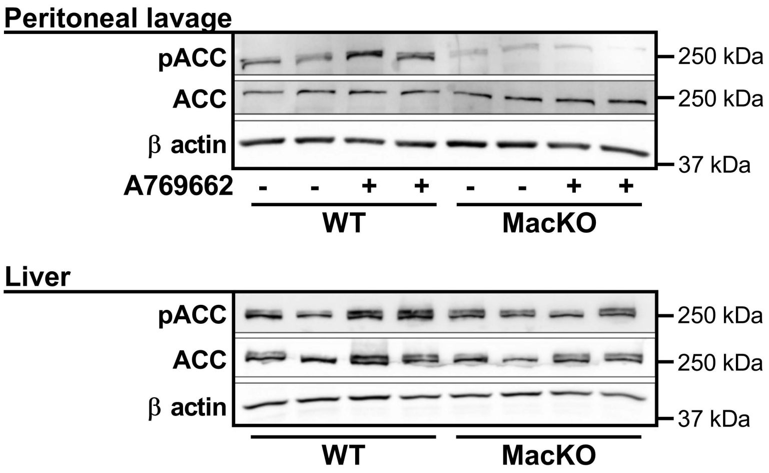

Supplementary Figure S1. Validation of myeloid AMPK-deficiency. Representative immunoblot depicting AMPK-specific signaling to ACC at S79 in peritoneal isolated cells (top) and liver (bottom). All mice received an I.P. injection of 3% thioglycolate four days prior to the isolation of cells from the peritoneal cavity and liver tissue. Peritoneal cells were harvested and incubated ± 100 μM A-769662 for 5 hours, whereas the liver was removed and probed without treatment. Total and phosphorylated ACC were assessed from duplicate gels.

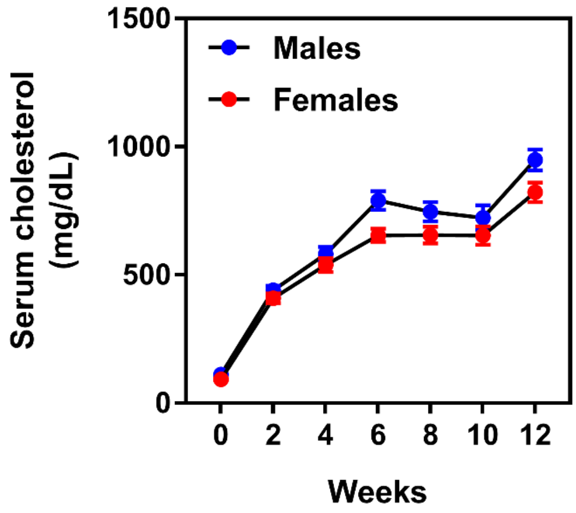

Supplementary Figure S2. WD-fed mice infected with PCSK9-AAV become hypercholesterolemic. Total blood cholesterol was measured weekly and shown as a pooled, representative group of male and female mice. Each data point represents the mean value from one animal ± SEM (n = 9-16/group).

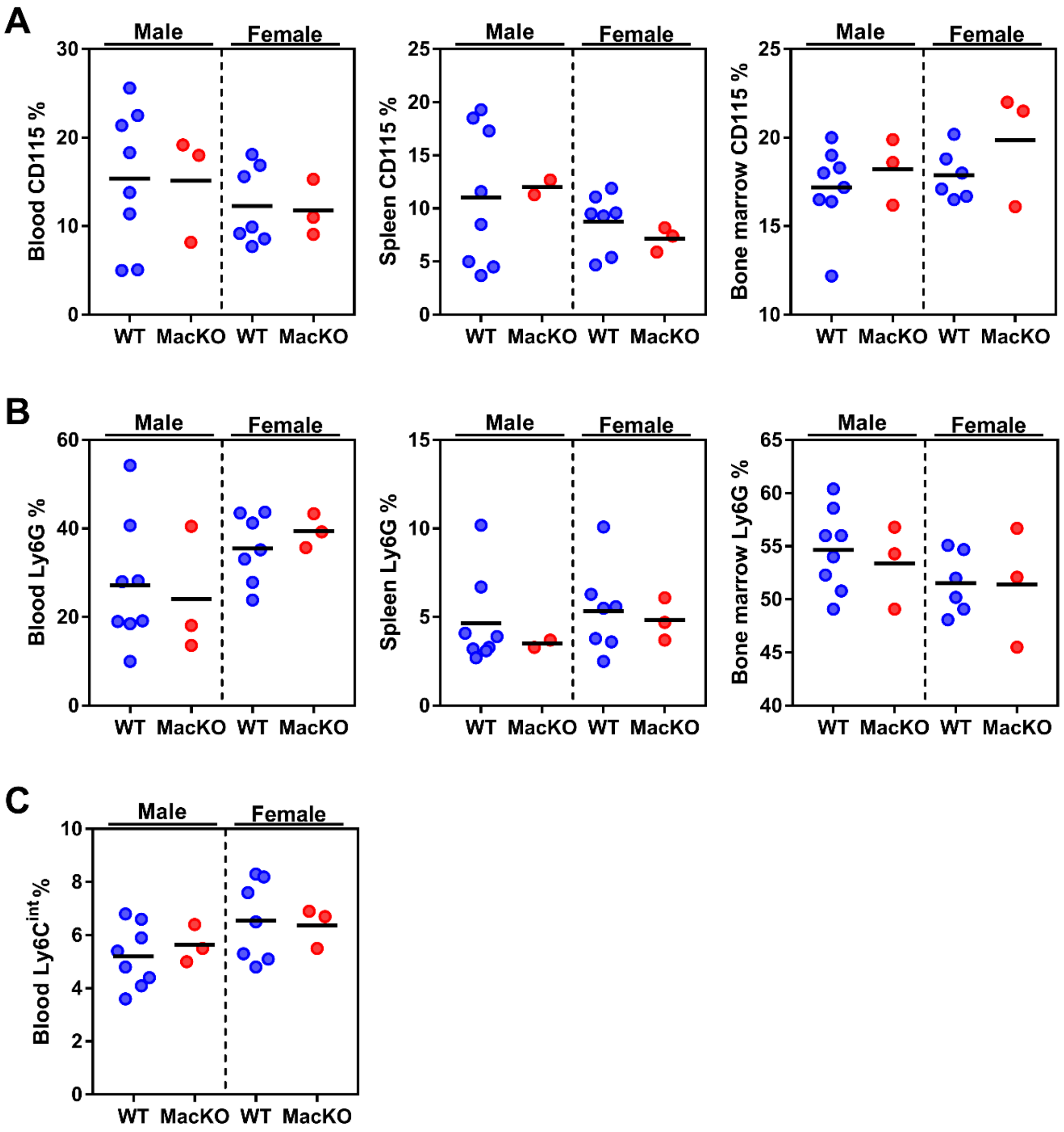

Supplementary Figure S3. Myeloid AMPK does not alter CD115 and Ly6G expressing cell populations. WT and MacKO male and female mice were injected with the PCSK9-AAV and fed a WD for 12 weeks. Cells expressing (A) CD115 and (B) Ly6G were quantified from the blood, spleen, and bone marrow. (C) Percentage of Ly6C^{int} expressing cells in circulation. Each data point represents the mean value from one animal (n = 2-8/group; a MacKO sample from the spleen was removed due to improper staining).

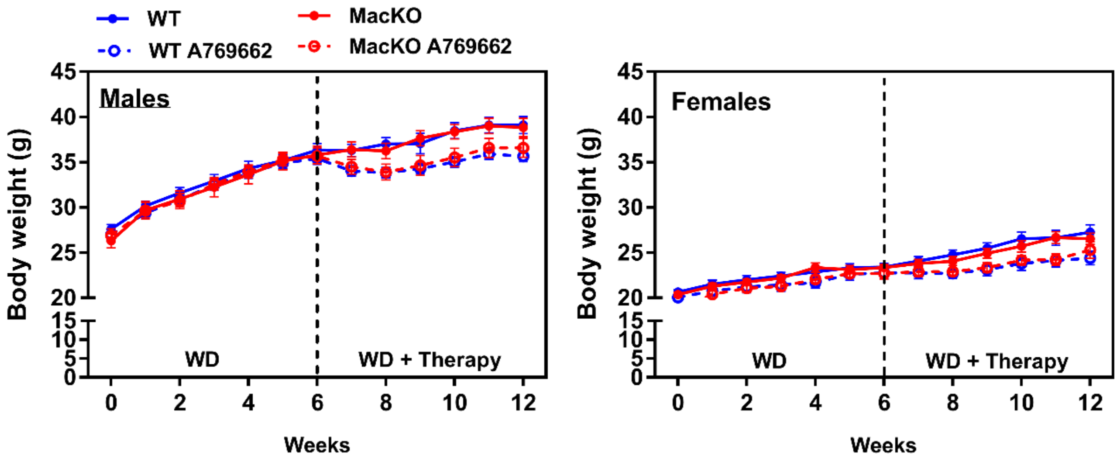

Supplementary Figure S4. Weekly mouse weights were monitored post-PCSK9-AAV injection for (A) male and (B) female WD-fed mice. At 6-weeks of HF-feeding mice were placed into groups and treated daily with either 30 mg/kg A-769662 or PBS control (I.P). Each data point represents the mean value from one animal ± SEM (n = 9-16/group).

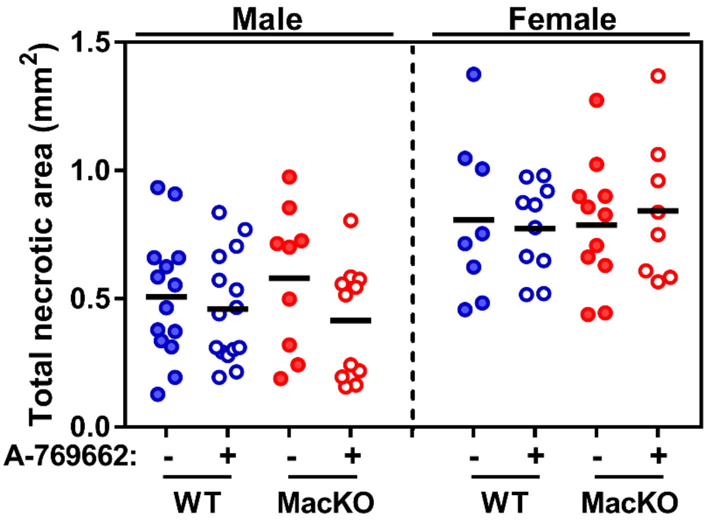

Supplementary Figure S5. The total necrotic area (denoted as white space absent of cellularity) was assessed in the lesions of male and female mice. Each data point represents the mean value from one animal (n = 8-15/group).

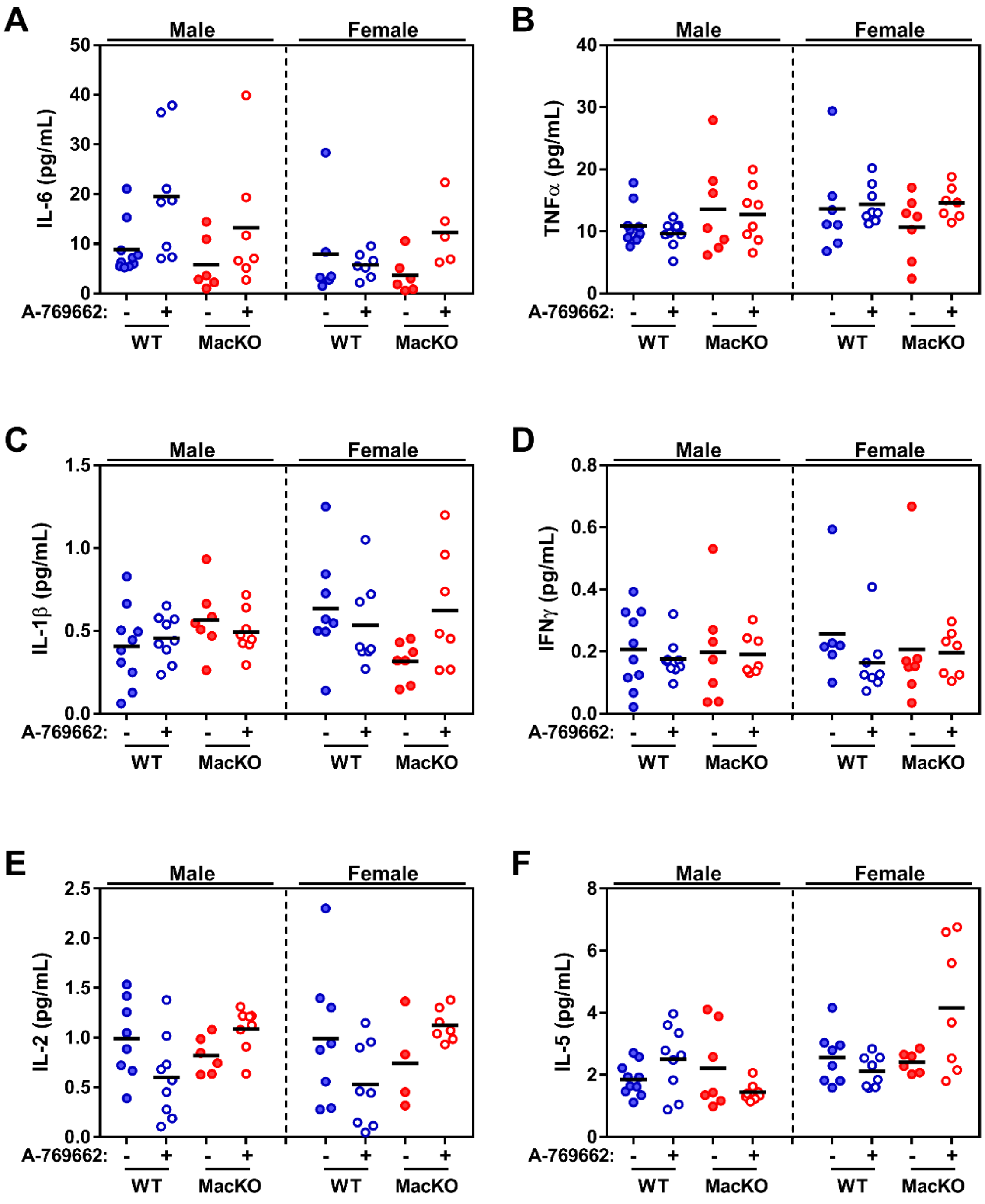

Supplementary Figure S6. Myeloid AMPK signaling does not alter systemic cytokine levels. Quantification of circulating inflammatory cytokines (A) IL-6, (B) TNFα, (C) IL-1β, (D) IFNγ, (E) IL-2, and (F) IL-5 was performed on endpoint serum samples obtained during tissue harvest. Each data point represents the value from one animal (n = 4-10/group; certain samples were below the detectable levels for select cytokines).

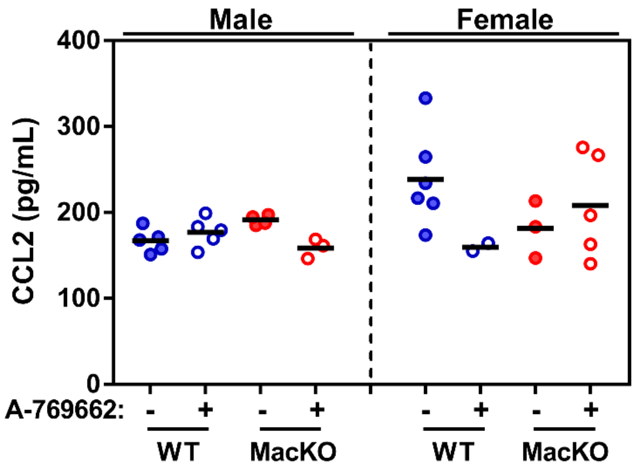

Supplementary Figure S7. Circulating levels of MCP1 measured from endpoint serum obtained during tissue harvest. Each data point represents the value from one animal (n = 2-6/group; certain samples were below the detectable levels for select cytokines).

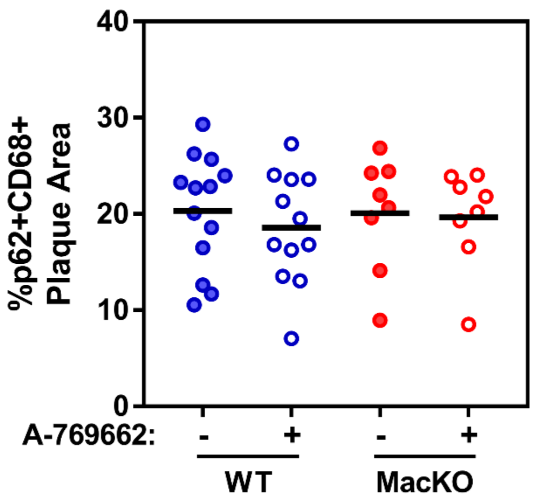

Supplementary Figure S8. Myeloid AMPK signaling does not influence markers of lesion autophagy. Quantification of p62 expression within CD68-positive cells by immunofluorescence in lesions from male mice.
